# Growth and Neurodevelopmental Outcome in Preterm LBW Infants with Sepsis in India: A Prospective Cohort

**DOI:** 10.1155/2018/5735632

**Published:** 2018-02-21

**Authors:** Sunil J. Pawar, Tejopratap Oleti, Siluvery Bharathi, Shyamsunder Tipparaju, Ershad Mustafa

**Affiliations:** ^1^Durgabai Deshmukh Hospital and Research Center, Hyderabad, India; ^2^Fernandez Hospital, Hyderabad, India; ^3^Thumbay Hospital New Life, Hyderabad, India

## Abstract

**Objective:**

Neonatal sepsis is associated with abnormal neurodevelopmental outcomes but not with poor growth at 9 to 15 months of corrected age in LBW infants.

**Design, Setting, and Participants:**

This is a prospective cohort study involving 128 eligible preterm low-birth-weight (LBW) infants admitted during the period of 2013-2014 to the Durgabai Deshmukh Hospital and Research Center. All patients were followed up in the outpatient Department of Pediatrics. They were divided into the sepsis and nonsepsis group.

**Results:**

A total of 94 infants were evaluated (40 in sepsis and 54 in nonsepsis group). At the age of 9–15 months, low-birth-weight infants with neonatal sepsis had an increased risk of neurodevelopmental disorders (67.5 versus 20.3%; RR: 3.31 (1.87–5.85)). There is no statistically significant difference in the growth outcomes.

**Conclusion:**

Neonatal infections are associated with the abnormal neurodevelopmental outcomes in LBW infants but there was no significant difference at growth outcome at 9 to 15 months of corrected age between both groups.

## 1. Introduction

Low-birth-weight (LBW) infants are at risk for poor growth and neurodevelopment outcome. In India, few studies are done showing the outcome between the very-low-birth-weight (VLBW) infants [1–3]. In India, about 33% of babies are born with low birth weight. Though a small percentage of these children develop cerebral palsy or mental retardation, long-term follow-up studies have shown mild problems in cognition, adjustment, and behaviour [4, 5] in early adolescence.

In year 2010, 15 million infants were born preterm worldwide. Out of these, 13 million were survived and 2.7% had moderate-to-severe neurodevelopment impairment [6]. Intrauterine infections are also associated with cerebral white matter injury and subsequent neurodevelopmental impairment [7]. The incidence of neonatal sepsis according to the data from National Neonatal Perinatal Database (NNPD, 2002-03) is 30 per 1000 live births. Septicemia was the commonest clinical category with an incidence of 23 per 1000 live births while the incidence of meningitis was reported to be 3 per 1000 live births.

Many LBW and ELBW infants have at least 1 episode of early-onset or late-onset infection during their initial hospital stay. Neonatal infections and necrotizing enterocolitis (NEC) have been linked with an increased risk of neurodevelopment impairment in LBW survivors [1, 8]. However, the prognostic importance of infection relative to the other neonatal morbidities remains uncertain. Many of these studies looked at the effect of sepsis on neurodevelopmental outcome as a secondary analysis. We undertook this study to examine whether adding infection improves the prediction of poor outcomes in LBW infants at 9 to 15 months of corrected age.

## 2. Methods

It is a prospective observational cohort study which is done in the year of January 2013 to December 2014 in Durgabai Deshmukh Hospital and Research Center, Hyderabad, India. All preterm LBW neonates with gestation ≤ 37 weeks born between January 2013 and December 2014 and discharged from the hospital were eligible for the study. Parental consent was obtained at the time of visit to the hospital for enrollment into the study for the long-term follow-up. The study was approved by the institute Ethical Committee. Infants with major congenital malformations, severe perinatal asphyxia (5 min Apgar < 4 and/or cord Ph < 7.0), and postneonatal meningitis or other CNS infections were excluded. A convenient sample size of 40 was taken due to financial and academic constraints. The study was continued till we got 40 neonates with sepsis.

A total of 120 eligible preterm LBW infants (less than 37 weeks and birth weight ≤ 2500 grams) were admitted in the hospital. Five infants died in the NICU before discharge. Five babies had 5 min Apgar ≤ 5. Two infants were diagnosed to have meningitis and 2 infants required syndrome evaluation. Thus, a total of 106 infants were discharged alive from the NICU and were eligible for long-term follow-up. Of these, 44 babies had screen and/or culture positive sepsis and 62 babies were control group. After 9 to 15 months of corrected age 40 infants in sepsis and 54 infants in control group were evaluated. Four babies in the sepsis group and 8 babies in the control group are lost to follow-up.

A total of 94 preterm infants with birth weight ≤ 2500 grams were available for analysis of outcome (40 in sepsis and 54 in nonsepsis group). All patients were born with less than 37 weeks of gestational age (according to ultrasound fetal measurements at the beginning of pregnancy or last menstrual period or modified Ballard score in absence of the former two). These infants were followed up in a neurodevelopment center at the outpatient unit of the Department of Pediatrics.

### 2.1. Primary Outcomes

#### 2.1.1. Growth

**Figure 1:**
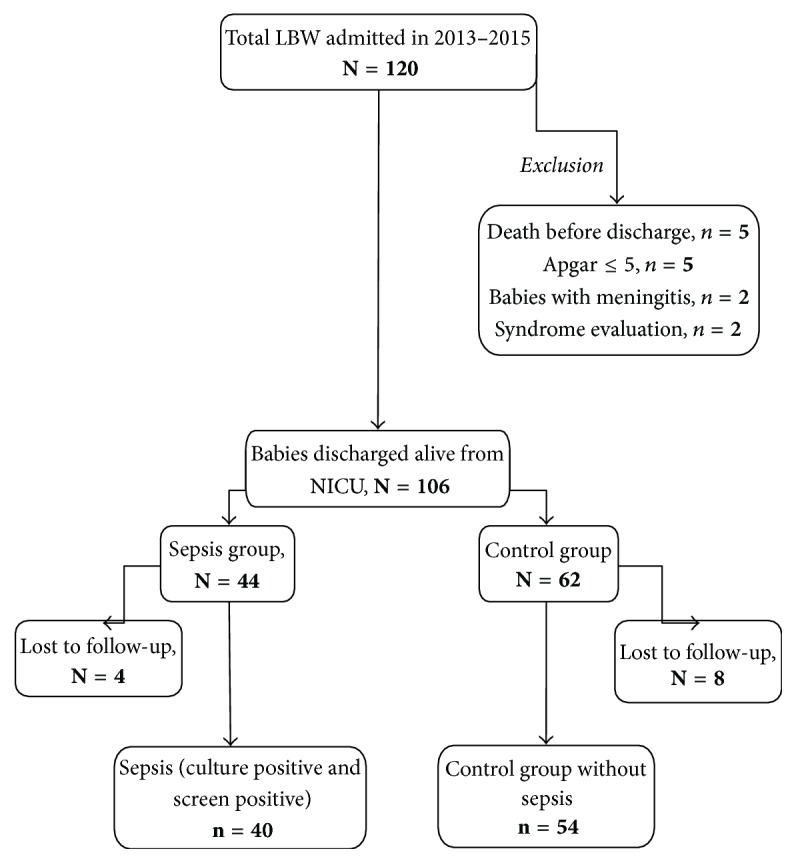


Growth is measured and depicted in terms of weight, length, and head circumference. Growth below the 5th centile when plotted on the Indian Academy of Pediatrics charts was considered as abnormal. For international comparison, growth below the 5th centile when plotted on the CDC functional growth charts was considered as abnormal (see Figure 1).

#### 2.1.2. Neurosensory/Neurodevelopmental Outcomes

*Cerebral Palsy (CP)*. It is a group of permanent, but not unchanging, disorders of movement and/or posture and of motor function, which are due to a nonprogressive interference, lesion, or abnormality of the developing/immature brain as defined by Surveillance of Cerebral Palsy in Europe (SCPE) definition. CP is defined by the presence of any one of the following: hemiplegia or diplegia or quadriplegia and/or presence of dystonia as assessed during neurological examination. 

*Visual Impairment*. It is defined as blindness in one or both eyes or need for corrective lenses. 

*Hearing Impairment*. It is defined as requirement of hearing aids in one or both ears. 

*Developmental Delay*. It is defined as more than 2 delays in any sector on the Denver Developmental Screening Test II (DDST II).

### 2.2. Secondary Outcomes at 9 to 15 Months of Corrected Age

*Tone Abnormalities*. They are abnormal tone of any limb, either hypotonia or hypertonia requiring physiotherapy without gait abnormality and not qualifying for the definition of cerebral palsy.

*Osteopenia of Prematurity*. It is defined as the presence of clinical features of rickets like Harrison sulcus, frontal bossing, wide anterior fontanel, and/or widening of wrist along with X-ray or biochemical changes (Alkaline phosphatase > 2.5 times the upper limit of normal and serum calcium < 8 mg/dl). 

*Hyperreactive Airway Disease*. It is considered when there were at least two documented episodes of wheezing requiring inhaled steroids or bronchodilators with/without systemic steroids. 

*Hospital Readmissions during Infancy*. It is one or more admissions after discharge from the hospital for any illness.

### 2.3. Data Collection

Gestational age was determined by first-trimester ultrasound scan. When the scan was not available it was based on mother's last menstrual period. The antenatal and perinatal details of enrolled infants were collected retrospectively from case files. This data included maternal demographic and clinical details and pregnancy related illnesses, antenatal steroid administration, and mode of delivery.

### 2.4. Neonatal Data

Relevant neonatal data of all LBW babies including morbidity and mortality during the hospital stay was recorded in a predesigned proforma at the time of discharge from the hospital. Recorded data included birth weight, sex, and the need for resuscitation at birth and Apgar scores at 1 and 5 minutes. Incidence of Respiratory Distress Syndrome (RDS), Patent Ductus Arteriosus (PDA) clinical presentation or echocardiography proven for hemodynamically significant cases [9], sepsis (blood, Cerebrospinal Fluid (CSF) culture positive, or septic screen positive), necrotizing enterocolitis (NEC) stage II and above, metabolic derangements like hypoglycemia, electrolyte disturbances, apneas, jaundice and presence of Intraventricular Hemorrhage (IVH), Retinopathy of Prematurity (ROP), and Broncho Pulmonary Dysplasia (BPD).

Sepsis is defined as having clinical suspicion with elevated White Blood Cells (WBC) count with predominance of immature granulocyte, polymorphonuclear cells (PMN) counts, depressed total White Blood Cell (WBC) count (<5000/mm^3^), and Absolute Neutrophil Count (ANC) (<1000/mm^3^) and an Immature-to-Total neutrophil count (I : T ratio) > 0.2 with or without culture positivity. Elevated C reactive protein (CRP) (>10 mg/dL) is also included as marker of screen positive sepsis. The babies without culture positivity will be considered as screen positive sepsis.

### 2.5. Follow-Up Data: Data on Growth and Neurodevelopment

#### 2.5.1. Period of Follow-Up

All infants discharged alive from the NICU were followed up periodically in the high risk neurodevelopment follow-up clinic. All enrolled infants were followed up weekly/biweekly till they were 40 weeks of gestational age, and then at 3, 6, and 9 months and within 15 months of corrected age.

#### 2.5.2. Retinal Examination

Retina examination for presence of Retinopathy of Prematurity was done by an ophthalmologist periodically from 3 weeks of postnatal life till resolution of ROP or complete vascularity. At 40 weeks of corrected gestational age, each infant underwent a Neurosonogram (NSG) and a Brain Stem Evoked Response Audiometry (BERA). At each visit babies were evaluated for growth and neurodevelopmental outcomes.

#### 2.5.3. Weight and Head Circumference

At each visit growth was evaluated by measuring the weight and head circumference and length. Weight was recorded by an electronic weighing scale corrected up to 2 decimals. Length was measured using an Infantometer by extending the legs and keeping the ankle in neutral position. Head circumference was measured with a nonstretchable tape.

#### 2.5.4. Neurological Assessment

Neurological assessment was done by Amiel Tison method. Developmental assessment was done using the Denver Development Screening Test II (DDST II) which assesses development in gross and fine motor, language, and social adaptive skills.

### 2.6. Statistical Analysis

All the outcome variables were compared between the study and each of the control groups separately. Statistical analysis was performed by using the Statistical Package for the Social Sciences (Version 19.0 for Windows, SPSS Inc., Chicago, IL). Chi square test was used for categorical variables and independent Student *t*-test was used for continuous variables. Predictor variables associated with abnormal outcomes were also studied. Relative risks (95% CI) were calculated for primary and secondary outcomes.

## 3. Results

### 3.1. Baseline Variables of the Study Group

Baseline data for 94 infants were included in the study; mean gestational age at birth was “SD + 2.03” weeks. Eighty-one (86.17%) infants were born through emergency caesarian section and 13 (13.82%) were born by spontaneous normal vaginal delivery. Eighty-one (86.17%) infants were given course of antenatal steroid. Seventy-seven (81.91%) were products of singleton pregnancies. Sixty-one (64.89%) infants were complicated by maternal hypertension. Oligohydramnios was present in 27 (28.72%) pregnancies, and seventeen (18.08%) babies were IUGR during pregnancy. Twenty-one (22.34%) infants were delivered prematurely because of preterm labor and 19 (20.21%) infants' mothers had preterm premature rupture of membrane. The above data is presented in Table 1(a).

### 3.2. Baseline Variables of the Neonatal Data

Of 94 eligible LBW infants who were discharged alive from the hospital, median birth weight was 1883 grams (IQR1560–2360), and 46 (48.93%) were female infants. Fourteen infants (14.89%) had birth asphyxia (but Apgar > 5 at 5 min) requiring resuscitation, and 41 (43.61%) infants were diagnosed to have HMD. Fifty-four (57.44%) infants and 21 (22.34%) infants required CPAP and ventilator support during their NICU stay due to respiratory distress, respectively. Nineteen (20.21%) infants had NEC, 48 infants (51.06%) had features of shock requiring fluid bolus or inotropes support, 9 infants (9.57%) had hypoglycemia requiring intravenous dextrose infusion, 8 infants (8.5%) had seizures, and 14 infants (14.89%) received at least one blood transfusion for anemia. 11 infants (11.70%) were diagnosed to have clinical or 2D-Echo proven PDA, and, of these, 7 infants were treated with Indomethacin or oral Ibuprofen for PDA closure. Forty babies (42.55%) were diagnosed to have sepsis. Among them culture positive was seen in 24 infants (25.53%) and only screen positive sepsis was present in 16 infants (17.02%). The above data is presented in Table 1(b).

### 3.3. Baseline Variables of Infants at the Time of Discharge

Among the surviving infants (*n* = 94) the mean time to achieve full enteral feeds in the study group was 7.88 (SD ± 3.11) days. The mean duration of hospital stay was 17.67 (SD ± 10.63). The mean weight, mean head circumference, and mean length at discharge were 1999 (SD ± 114), 29.51 (SD ± 1.72), and 39.15 (SD ± 1.80), respectively. Seventeen infants (18.08%) had Retinopathy of Prematurity and of these 4 (23.52%) required laser therapy.

Twelve infants (12.76%) had abnormal BERA, and of these 4 (33%) required hearing aids. 30 (31.91%) babies had abnormal NSG at term corrected age. The above data is presented in Table 1(c).

### 3.4. Comparison between Sepsis Group and Control Group

Of the total 94 infants followed up at corrected age of 9 to 15 months, 40 infants had sepsis and 54 infants were without sepsis as control group. On comparing the sepsis group with control group the mean gestational age and mean birth weight were equal in sepsis group (mean GA 30.275 (SD ± 2.20) WK versus mean 31.25 (SD ± 1.86) Wk and mean birth wt 1706 (IQR1560–1880) gms versus mean birth wt 2060 (IQR 1650–2360) gms, resp.). The sex ratio and maternal variables such as PIH (67.5% versus 62.9%), gestational DM (15% versus 9.2%), hypothyroid (15% versus 18.1%), and APH (2.5% versus 7.4%) were similar in both the groups. The incidences of preterm PPROM (27.5% versus 14.8%), preterm labor (15% versus 27.7%), and antenatal oligohydramnios (32.5% versus 25.92%) were also similar in both the groups. Antenatal steroid coverage (90% versus 83.30%) and mode of delivery by LSCS were also similar in both groups (87.5% versus 85.18%). The resuscitation required in the septic group is significantly higher (30% versus 7.4%) as compared to control group. These data are presented in Tables 2(a) and 2(b).

### 3.5. Comparison of NICU Morbidities in Sepsis and Control Group

Almost most of the neonatal morbidities were significantly higher in sepsis group, except HMD, CPAP, and HFO which were comparable in both groups. Tables 2(c) and 2(d) represent NICU morbidities between both groups. On comparing NICU morbidities anemia, PDA, CLD, discharge weight, discharge OFC, discharge length, ROP abnormal BERA, and abnormal NSG are significantly higher in the sepsis group as compared to the control group. Other variables, like days to full feed and hospital stay are comparable in both the groups.

### 3.6. Follow-Up Data of Study Population

During infancy, at 6 months of chronological age, 44 infants (46.80%) continued to be on exclusive breastfeeds, and 36 (38.29%) infants were on mixed feeds (breast feeds and formula milk). The median time of weaning or starting of solid supplements was 5 months of chronological age. In the majority of infants the weaning diet was a mixture of homemade diet and commercial weaning foods available in the market. The above data is presented in Table 3.

### 3.7. Primary and Secondary Outcome

At the age of 9–15 months a total of 38 infants (40.42%) had abnormal neurological outcome, 7 (7.44%) had cerebral palsy, and 4 babies (4.2%) had deafness requiring hearing aids. 18 infants (19.1%) failed DDST II, 6 infants (6.3%) had squint, and 4 infants (4.2%) had blindness.

### 3.8. Growth Outcome

**Figure 2:**
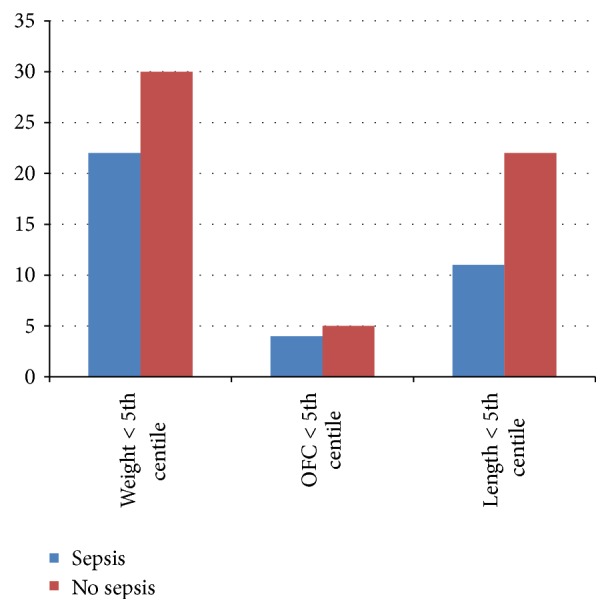
Bar diagram showing growth outcome according to CDC chart.

**Figure 3:**
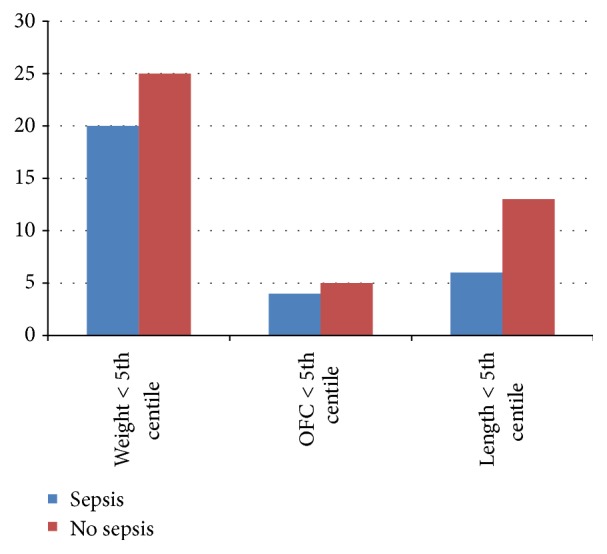
Bar diagram showing growth outcome according to IAP chart.

When plotted on the IAP chart, 45 infants (47.87%) had weight less than the 5th centile. Nine infants (9.5%) and 19 infants (20.21%) had head circumference and length less than the 5th centile, respectively (Figure 2). When plotted on the CDC 2000 growth charts, the growth patterns showed a similar trend. Fifty-two infants (55.31%) had weight less than the 5th centile. Nine infants (9.5%) had a head circumference below the 5th centile at 9 to 15 months of corrected age. Thirty-three (39.36%) infants had length below the 5th centile. There was no significant difference at growth outcome at 9 to 15 months of corrected age between sepsis group and the control group (*p* < 0.72). Also incidences of small head (<3rd centile in WHO growth charts) and short stature (<than 2 SD on WHO growth charts) in both the groups were comparable (Figure 3).

### 3.9. Neurological Outcome

The abnormal neurological outcome constitutes the infants with cerebral palsy, infants requiring hearing aids, infants who failed DDST, and infants who had squint and blindness. Number of infants evaluated between 9 and 12 months in sepsis and control group was 26 (65%) and 33 (61.1%), respectively, and infants evaluated between 12 and 15 months in sepsis and control group were 14 (35%) and 21 (38.8%), respectively.

**Figure 4:**
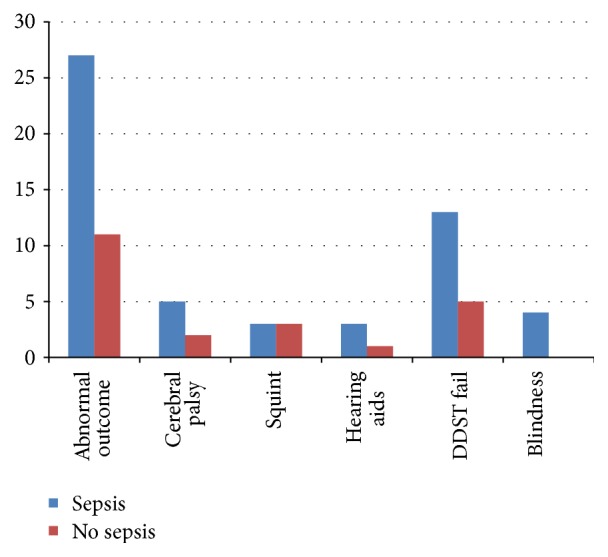
Abnormal outcome between sepsis group and the control group.

Comparing the outcomes between the two groups the outcome of abnormal neurological examination was significantly more in sepsis group than in control group (*p* < 0.001). The data is presented in Table 4 and Figure 4. The incidence of cerebral palsy was more in sepsis group but did not reach statistical significance. Incidences of tone abnormality not qualifying for cerebral palsy, squint, and osteopenia were similar between the two groups. The incidence of DDST II failure is more in the sepsis group as compared to control group.

## 4. Discussion

Our study showed that preterm LBW infants with sepsis had a significantly higher incidence of abnormal neurodevelopmental outcomes at the corrected age of 9–15 months. However we did not do logistic regression to rule out the effect of other neonatal comorbidities on the long-term outcome in view of small size of study cohort. Similar to our results were those found in a prospective observational study conducted by Manikyamba et al. [10] in LBW babies admitted in NICU with complications (HMD, sepsis, apnea, and hyperbilirubinemia). Surviving babies were followed up for one year to monitor their growth and development. Out of 700 LBW babies 73.8% were preterm and 26.2% were term IUGR. Overall mortality was 23.7%. Major causes of mortality were RDS and sepsis among preterm babies and sepsis and birth asphyxia among term IUGR babies. Out of 534 babies, 31% had poor catch-up growth and 22% had developmental delay.

In a systematic review and meta-analysis, comparing neurodevelopmental outcomes in VLBW infants exposed to culture-proven sepsis in the neonatal period with similar infants without sepsis, 15,331 infants were evaluated. Sepsis in VLBW infants was associated with an increased risk of one or more long-term neurodevelopmental impairments (odds ratio (OR) 2.09; 95% confidence interval (CI) 1.65 to 2.65) including cerebral palsy (CP; OR 2.09; 95% CI 1.78 to 2.45). Heterogeneity (*I*(2) = 36.9%; *p* = 0.06) between the studies was significant and related to variations in patient characteristics, causative pathogens, and follow-up methods [11].

Mukhopadhyay et al. [3] conducted a prospective study from 2007 to 2009 on 101 VLBW babies, of which 3% babies had cerebral palsy and 3% had mild hypotonia, 11% had gross motor delay, and 8% had language abnormality. Recently, the NICHD Neonatal Research Network reported that while 16% of ELBW infants were small for gestational age at birth, by 36 weeks of PCA, 89% had growth failure [12]. Furthermore, by 18 to 22 months of corrected age, 40% still were at less than the 10th percentile for weight, length, and head circumference [13]. Stoll et al. demonstrates that infection affects weight and head circumference at both 36 weeks of PCA and 18 to 22 months of corrected gestational age. Of greatest concern was their (Stoll et al.) finding that infants with neonatal infections were significantly more likely to have poor head growth. The long-term impact of impaired growth during and following neonatal infections deserves further study. Our study showed that infants with the sepsis were significantly growth retarded (wt 2 SD) at 9 to 15 months of corrected age (*p*  0.014) as compared to control group; however in comparison with other studies there was no significant difference in OFC and length at 9 to 15 months as our study did not have power to pick up the difference.

The premature infants are at risk of major and minor deficits such as cerebral palsy, cognitive and speech delays, motor and visual deficits, psychosocial and behavioural disorders and dysfunction at school. Soleimani et al. [14] conducted an observational study in preterm babies and observed cerebral palsy especially spastic diplegia, intellectual disability, and visual and hearing impairments are the main neurodevelopmental disorders associated with prematurity and there is increasing evidence of sustained adverse outcomes into school age and adolescence, for preterm infants. We found that there is a significant delay in neurodevelopment in sepsis group compared to nonsepsis group at 9 to 15 months of corrected age (*p*  0.001) and also found that there is a gap between the high technology and quality of medical care and loss of or no neurodevelopmental care for high risk neonates and infants in NICUs by specialties and nursing of long-term neurodevelopmental outcome for high risk neonates. Modi et al. [15] evaluated very-low-birth-weight (VLBW) infants in their growth during NICU stay with a catch-up later during infancy. In comparison to Normal-Birth-Weight (NBW) infants, they continue to lag in their physical growth and neurodevelopment at 1 year of corrected age.

## 5. Conclusion

Neonatal sepsis are associated with abnormal neurodevelopmental outcomes but not with poor growth at 9 to 15 months of corrected age in LBW infants. However, these associations should be studied in different settings with larger sample sizes.

## Figures and Tables

**Table:** (a) Baseline variables of the studied patients

Variable	(*N* = 94)
Mean gestation (weeks)	30.76 (SD ± 2.03)
Singleton	77 (81.91%)
Primi (refers to first gestation)	42 (44.68%)
APH	05 (05.30%)
Maternal diabetes	09 (09.59%)
Maternal hypertension	61 (64.89%)
Maternal hypothyroid	16 (17.02%)
Maternal rupture of membranes	19 (20.21%)
Oligohydramnios	27 (28.72)%
Steroid covered	81 (86.17%)
Caesarian section	81 (86.17%)
Preterm labor	21 (22.34%)
IUGR	17 (18.08%)

APH: antepartum hemorrhage; IUGR: intrauterine growth retardation.

**Table:** (b) Baseline variables of the neonatal data

Variable	*n* = 94
Median birth weight (grams)	1883 (IQR 1560–2360)
Median head circumference at birth (cm)	28.5 (IQR 26–31)
Median length at birth (cm)	37.69 (IQR 34–40)
Sex males	48 (51%)
HMD	55 (58.51%)
CPAP	54 (57.44%)
Ventilation	21 (22.34%)
Sepsis	40 (42.55%)
Culture positive sepsis	24 (25.53%)
Screen positive sepsis	16 (17.02%)
NEC	19 (20.21%)
Shock	48 (51.06%)
Seizure	8 (8.5%)
Hypoglycemia	9 (9.57%)
Apnea	14 (14.89%)
Anemia transfusion	14 (14.89%)
PDA	11 (11.72%)

BPD: bronchopulmonary dysplasia; PDA: Patent Ductus Arteriosus; NEC: necrotizing enterocolitis; HFO: high frequency oscillatory ventilation; SIMV: synchronized intermittent mandatory ventilation; HMD: hyaline membrane disease; CPAP: continuous positive airway pressure.

**Table:** (c) Baseline variables of infants at the time of discharge

Variable	Total (*n* = 94)
Days to full feeds median (days)	7.88 (SD ± 3.11)
Duration of hospital stay (days)	17.67 (SD ± 10.63)
Discharge weight (grams)	1999 (SD ± 114)
Discharge head circumference (cm)	29.51 (SD ± 1.72)
Discharge length (cm)	39.15 (SD ± 1.80)
ROP	17 (18.08%)
ROP with LASER	4 (4.25%)
Abnormal BERA	12 (12.76%)
Abnormal NSG	30 (31.91%)

ROP: Retinopathy of Prematurity.

**Table:** (a) Comparison of basic variables between both studied groups

Variable	Sepsis	No sepsis	*p* value
Gestational age, mean (wk)	30.275 (SD ± 2.20)	31.22 (SD ± 1.86)	<0.02
Birth WT, median (gms)	1706 (IQR 1560–1880)	2060 (IQR 1650–2360)	<0.01
Birth OFC, median (cm)	27.7 (IQR 26–30)	29.57 (IQR 27–31)	<0.01
Birth length, median (cm)	36.9 (IQR 34–39)	38.48 (IQR 35–40)	<0.01
Male	23 (57.5%)	25 (46.2%)	0.28

WT: weight; OFC: occipitofrontal circumference.

**Table:** (b) Comparison of antenatal events between both studied groups

Variable	Sepsis (*N* = 40)	No sepsis (*N* = 54)	*p* value
Singleton	32 (80%)	45 (83.3%)	0.90
Primi	19 (47.5%)	25 (46.2%)	0.82
AGA	21 (52.7%)	28 (51.85%)	0.95
PIH	27 (67.5%)	34 (62.9%)	0.64
Diabetes	6 (15%)	5 (9.2%)	0.41
Hypothyroid	6 (15%)	10 (18.51%)	0.70
PPROM	11 (27.5%)	08 (14.81%)	0.13
Preterm labor	6 (15%)	15 (27.7%)	0.14
Oligohydramnios	13 (32.5%)	14 (25.92%)	0.49
APH	1 (2.5%)	4 (7.4%)	0.29
Steroid covered	36 (90%)	45 (83.3%)	0.35
LSCS	35 (87.5%)	46 (85.18%)	0.75
Resuscitation at birth	12 (30%)	4 (7.4%)	<0.001

AGA: appropriate for gestational age; PIH: pregnancy induced hypertension; PPROM: preterm prelabor rupture of membranes; APH; antepartum hemorrhage; LSCS: lower segment caesarian section.

**Table:** (c) Comparison of NICU morbidities between both studied groups

Variable	Sepsis (*n* = 40)	No sepsis (*n* = 54)	*p* value
HMD	21 (52.5%)	20 (37.03%)	0.13
CPAP	26 (65%)	28 (51.8%)	0.30
Surfactant	22 (55%)	15 (27.7%)	0.007
SIMV	13 (32.5%)	5 (9.2%)	<0.001
HFO	6 (15%)	5 (9.2%)	0.39
NEC	16 (40%)	3 (5.5%)	<0.001
Shock	35 (87.5%)	13 (24.07%)	<0.001
Exchange transfusion	19 (47.5%)	00	<0.001
Seizure	6 (15%)	2 (3.7%)	0.05
Hypoglycemia	8 (20%)	1 (1.8%)	<0.001
Apnea	13 (32.5%)	1 (1.8%)	<0.001
Anemia transfusion	8 (20%)	6 (11.11%)	0.22
PDA	9 (22.5%)	2 (3.7%)	0.004
BPD	7 (17.5%)	3 (5.55%)	0.06

BPD: bronchopulmonary dysplasia; PDA: Patent Ductus Arteriosus; NEC: necrotizing enterocolitis; HFO: high frequency oscillatory ventilation; SIMV: synchronized intermittent mandatory ventilation; HMD: hyaline membrane disease; CPAP: continuous positive airway pressure.

**Table:** (d) Comparison of NICU variables between both studied groups

Variable	Sepsis (*n* = 40)	No sepsis (*n* = 54)	*p* value
Discharge WT, mean (gms)	1902 (SD ± 90.55)	2097 (SD ± 137)	0.01
Discharge OFC, mean (cm)	28.95 (SD ± 2.366)	30.07 (SD ± 1.078)	0.01
Discharge LT, mean (cm)	38.8 (SD ± 1.70)	39.65 (SD ± 1.90)	0.01
Days to full feeds, mean (days)	7.6 (SD ± 2.92)	8.1 (SD ± 3.30)	0.39
Hospital stay, mean	16.8 (SD ± 6.31)	18.48 (SD ± 8.64)	0.32
BERA (abnormal)	10 (25%)	2 (3.7%)	0.001
ROP	10 (25%)	7 (12.9%)	0.001
NSG (abnormal)	25 (62.5%)	5 (9.2%)	<0.001

WT: weight; OFC: occipitofrontal circumference; LT: length; BERA: brainstem evoked response audiometry; ROP: Retinopathy of Prematurity; NSG: Neurosonogram.

**Table 3:** Follow-up data of study population.

Variable	Measure
Feeding at 6 months	
Only BF	37 (37.2%)
Only formula	70 (74.4%)
Mixed	50 (53.19%)

Weaning started	5 (4–6)
Median (IQR) (months) Weaning started after	
4 to 6 months	71 (75.53%)
>6 months	24 (25.53%)

Weaning diet	
Only commercial food	40 (42.55%)
Home foods	04 (4.255%)
Mixed diet	51 (54.25%)

**Table 4:** Neurodevelopmental outcomes in both studied groups.

Outcome	Sepsis *n* (%)	No sepsis *n* (%)	RR (95% CI)	*p* value
Abnormal outcome	27/40 (67.5%)	11/54 (20.37%)	3.31 (1.87–5.85)	<0.001
Cerebral palsy	5/40 (12.5%)	2/54 (3.7%)	3.37 (0.69–16.5)	0.11
Squint	3/40 (7.5%)	3/54 (5.5%)	1.35 (0.28–6.34)	0.70
Hearing aids	3/40 (7.5%)	1/54 (1.8%)	4.05 (0.43–37.5)	0.18
DDST fail	13/40 (32.5%)	5/54 (9.2%)	3.51 (1.36–9.04)	0.001
Blindness	4/40 (10%)	00	NA	0.01
Tone abnormalities	4/40 (10%)	1/54 (1.8%)	5.4 (0.62–46.49)	0.083

DDST: Denver development screening test.

## Additional Points

*Merits of Our Study. *Our study is done in a low resource setting and evaluated neonatal sepsis as the primary factor for association with abnormal neurodevelopmental outcomes in preterm LBW infants. The study had good follow-up rate and included all relevant parameters.* Limitations of Our Study*. Our study did not have power to pick up the difference in CP alone or head growth. Neurodevelopment assessment was done with DDST II due to logistic reason, even though BSID is considered a better tool. Logistic regression could not be done for evaluating the independent association for abnormal neurodevelopment outcome as the number of infants with abnormal outcome is limited.

## References

[1] Chaudhari S., Otiv M., Chitale A., Pandit A., Hoge M. (2004). Pune Low Birth Weight Study - Cognitive Abilities and Educational Performance at Twelve Years. *Indian Pediatrics*.

[2] Bhargava S. K., Ramji S., Srivastava U. (1995). Growth and sexual maturation of low birth weight children: a 14 year follow up. *Indian Pediatrics*.

[3] Mukhopadhyay K., Malhi P., Mahajan R., Narang A. (2010). Neurodevelopmental and behavioral outcome of very low birth weight babies at corrected age of 2 years. *The Indian Journal of Pediatrics*.

[4] Hack M., Klein N. K., Taylor H. G. (1995). Long-Term Developmental Outcomes of Low Birth Weight Infants. *The Future of Children*.

[5] Hack M., Flannery D. J., Schluchter M., Cartar L., Borawski E., Klein N. (2002). Outcomes in Young Adulthood for Very-Low-Birth-Weight Infants. *Obstetrical & Gynecological Survey*.

[6] Robaina Castellanos G. R., Riesgo Rodríguez S. D. L. C. (2016). Neonatal sepsis and neurodevelopment in very low birth weight infants in Matanzas, Cuba 2006-2010: a prospective cohort study. *Medwave*.

[7] Nelson K. B., Willoughby R. E. (2002). Overview: Infection during pregnancy and neurologic outcome in the child. *Mental Retardation and Developmental Disabilities Research Reviews*.

[8] Chaudhari S., Otiv M., Khairnar B., Pandit A., Hoge M., Sayyad M. (2012). Pune low birth weight study - Growth from birth to adulthood. *Indian Pediatrics*.

[9] Pawar S., Sharma D., Murki S., Subramaniam S., Kandraju H. (2017). Construction of Ductal Diameter Centiles in the First 24 h of Life and Their Relation to Cerebral Blood Flow in Neonates Weighing Less Than 1250 g in the First 24 h of Life. *Journal of Tropical Pediatrics*.

[10] Manikyamba D., Madhavi N., Krishna Prasad A. (2015). Anitha, Morbidity and Mortality Profile of LBW Babies and Their Growth and Neurodevelopment Outcome at 1 year- NICU. *Scholars Journal of Applied Medical Sciences*.

[11] Alshaikh B., Yusuf K., Sauve R. (2013). Neurodevelopmental outcomes of very low birth weight infants with neonatal sepsis: Systematic review and meta-analysis. *Journal of Perinatology*.

[12] Dusick A. M., Poindexter B. B., Ehrenkranz R. A., Lemons J. A. (2003). Growth failure in the preterm infant: Can we catch up?. *Seminars in Perinatology*.

[13] Dusick A., Vohr B. R., Steichen J., Wright L. L., Mele L., Verter J. (1998). Factors affecting growth outcome at 18 months in extremely low birthweight (ELBW) infants. *Pediatric Research*.

[14] Soleimani F., Zaheri F., Abdi F. (2014). Long-term neurodevelopmental outcomes after preterm birth. *Iranian Red Crescent Medical Journal*.

[15] Modi M., Saluja S., Kler N. (2013). Growth and neurodevelopmental outcome of VLBW infants at 1 year corrected age. *Indian Pediatrics*.

